# Volumetric Light-Field Excitation

**DOI:** 10.1038/srep29193

**Published:** 2016-07-01

**Authors:** David C. Schedl, Oliver Bimber

**Affiliations:** 1 Institute of Computer Graphics, Johannes Kepler University, Linz, 4040, Austria

## Abstract

We explain how to concentrate light simultaneously at multiple selected volumetric positions by means of a 4D illumination light field. First, to select target objects, a 4D imaging light field is captured. A light field mask is then computed automatically for this selection to avoid illumination of the remaining areas. With one-photon illumination, simultaneous generation of complex volumetric light patterns becomes possible. As a full light-field can be captured and projected simultaneously at the desired exposure and excitation times, short readout and lighting durations are supported.

Fast optical volumetric recording, as required in numerous applications, still remains a challenge in microscopy. While regular scanning methods (e.g., confocal microscopy and its variants) can be too slow, specialized light-sheet scanning^1^, 3D imaging with spatial light modulators (SLMs)^2^, multi-focus microscopy^3^, and light-field microscopy (LFM)^4^ promise faster scanning rates.

LFM^5^ captures the 4D incident light by means of a microlens array (MLA) placed at the intermediate image plane of the imaging path, and thus supports the computation of a focal stack with a single sensor recording. The limited spatial resolution that is caused by simultaneously multiplexing 2D location and 2D direction information at the same image sensor can be increased computationally by 3D deconvolution^6^ and by using additional phase masks in the optical path^7^.

Besides fast readouts, precise controllable excitation is another requirement in recent microscopy techniques. In fluorescence microscopy, for instance, such methods are used to accurately illuminate only certain parts of a probe and to avoid out-of-focus excitation or even damaging the probe by photobleaching or phototoxicity. Spatially controllable excitation can be achieved by sequentially illuminating the sample with a focused beam while either moving the sample or scanning the beam with a mirror^8^. Multiple simultaneous beams can be generated with spatial light modulators (SLMs), such as digital micromirror devices^9^, or phase-modulators (PMs), such as liquid crystal PMs used for holographic projection^10^. The latter require coherent light produced by a laser light source.

One-photon excitation as discussed above, however, also excites the probe in the light cone above and below the focal plane, as illustrated in Fig. 1a, which makes controlled excitation within a volumetric probe impossible. Out-of-focus excitation can be avoided by two-photon techniques^11^,^12^,^13^,^14^, as shown in Fig. 1b. This allows deeper penetration in scattering media than simple one-photon methods, but limits simultaneous excitation to a single plane.

Various light-modulation approaches exist that strive for controlled, simultaneous excitation of multiple selected sample points within a volume. Combining two micromirror arrays in the illumination path of a microscope (one in the intermediate image plane and one in the back focal plane^15^,^16^), for instance, enables the generation of spatial-angular controlled light patterns to avoid out-of-focus excitation. However, the interplay of two 2D SLMs does not support arbitrary illumination patterns within a volume. Either angular sampling is constant for a spatial location, or spatial sampling is constant for a direction. Thus, an illumination pattern as shown in Fig. 1c cannot be achieved simultaneously, but requires sequential excitation.

Holographic projection with PMs^2^,^17^,^18^ overcomes this primary limitation. However, spatially varying diffraction efficiency and the presence of zero-order diffraction spots, ghosting, and intensity fluctuations (speckles) limit the excitation area and the spatial and temporal resolution of holographic projections^19^,^20^. Furthermore, coherent computer-generated holograms limit the types of illumination patterns that can be generated^21^.

We explain how to concentrate light simultaneously at multiple selected volumetric positions by means of a 4D illumination light field^22^. First, a 4D imaging light field is captured to select target objects. A light field mask is then computed automatically for this selection to avoid illumination of the remaining areas. For one-photon illumination, the simultaneous generation of complex volumetric illumination patterns, like the ones shown in Figs 1c and 7, becomes possible. In contrast to holographic projection approaches, light-field projection supports arbitrary patterns and non-coherent light sources, and retains the same sampling quality across the whole field of view. Since a full light field can be captured and projected simultaneously at the desired exposure and excitation times, short readout and illumination durations are supported.

## Masked Light-Field Illumination

**Figure 2:**
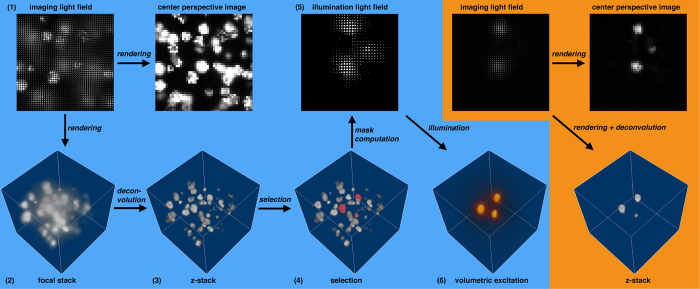
Volumetric light-field excitation principle (blue): (**1**) light-field imaging of probe under full illumination, (**2**) synthetic aperture rendering to obtain 3D focal stack, (**3**) 3D deconvolution to obtain 3D z-stack, (**4**) segmentation of selected regions, (**5**) computation of masked 4D light-field illumination from selection in z-stack, (**6**) projection of light-field illumination into the probe (colors indicate the concentration of light). Results with volumetric light-field excitation (orange): light-field imaging, synthetic aperture rendering and deconvolution to obtain z-stack and perspective images. In this experiment, 10–20 μm fluorescent microspheres and a water-immersion 60×/1.2 NA objective (see Table 1 for details) have been used. See also Supplementary Video 1.

Figure 2 illustrates the principle of volumetric excitation by means of masked light-field illumination using 10–20 μm fluorescent microspheres. With one sensor recording, we capture a 4D light field of the probe under full illumination and apply synthetic aperture rendering^23^,^24^ to compute a 3D focal stack or individual perspective images from it. The focal stack undergoes 3D deconvolution^25^ to obtain a defocus-free z-stack. Within the z-stack, we select parts of the probe (i.e., 3D-segmented microspheres, shown in red in our example) that are to be excited. From the selection in the z-stack, we then determine a 4D light-field mask that is projected simultaneously into the probe such that as much light as possible is concentrated at the volumetric positions of the selection while minimizing the illumination of other regions. Rerecording a light field using volumetric excitation shows selected regions in perspective images and z-stack (Fig. 2 orange).

Computing the masked light field that leads to the desired volumetric excitation pattern is the goal of the following optimization: Let *v*_*i*_ be the illumination reaching volumetric point *i, α*_*i*_ the transmission of light at *i*, and *r*_*j*_ the light intensity of light-field ray *j*, then *β*_*i*,*j*_ is the total transmission of ray *j* to volumetric point 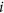 through all volumetric points on its path through the probe to *i: β*_*i*,*j*_ = *α*_1_ · *α*_2_ · … · *α*_*n*_, where volumetric points 1, 2, …, *n* are on the path of ray *j* to volumetric point *i*. The light transport for a given light field through a given volume can then be described as:


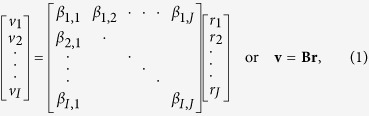


where *r* = [*r*_1_, *r*_2_, …, *r*_*J*_] is the vector of all light-field ray intensities, *v* = [*v*_1_, *v*_2_, …, *v*_*I*_] the vector of all light reaching each volumetric point, and **B** the transport matrix of all ray-to-point transmission coefficients *β*_*i*,*j*_ through the probe. With known transport matrix **B**, the vector **v** can be defined to contain the coefficient 1 for all volumetric sample points to be excited, and the coefficient 0 for all sample points not to be excited. Note that we assume 1 and 0 to correspond to the maximum and minimum brightness of the light source, respectively. Volumetric sample points whose illumination is irrelevant, such as carrier material (e.g., index matching gel), must not be included in **v**. Solving Eq. 1 for **r** results in an illumination light-field mask that maximizes irradiation in the selected points to be excited while minimizing the light in points that must not be excited. We apply the simultaneous algebraic reconstruction technique (SART)^26^ to solve Eq. 1.

For illumination light fields of *J* rays and volumes of *I* points, **B** is large and contains *I* × *J* coefficients. Despite being sparse, Eq. 1 is expensive to solve. For practical real-time applications, we approximate the illumination mask as follows:

We distinguish between three types of volumetric points: Those that should be excited (*E*, e.g., microspheres to be stimulated), those that should not be exited (*N*, e.g., microspheres not to be stimulated), and those for which illumination is irrelevant (*O*, e.g., carrier material such as an index-matching gel).

We then determine all light-field rays *R* that pass through points in *E* by raycasting the z-stack, and consider three general application-specific illumination strategies: (S1) All rays in *R* equal 1 (which corresponds to a full illumination along the rays). (S2) Rays in *R* equal 1 only if they do not pass through points in *N*. (S3) Rays in *R* equal 1 only if they do not pass through points in *N* before they pass through a point in *E*. All other rays of the illumination light field are set to 0 (no light).

**Figure 3:**
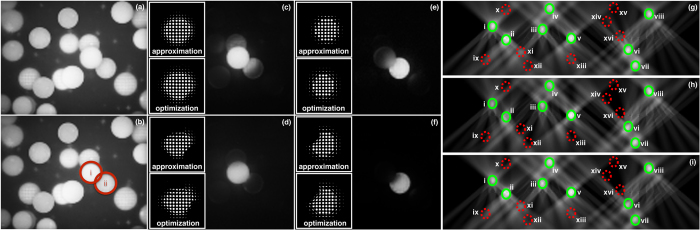
Example of excitation with computed light-field masks. (**a**,**b**) Two occluding microspheres i and ii; (**c**,**d**) i is to be excited, while ii is to remain unexcited; (**e**,**f**) ii is to be excited, while i is to remain unexcited; (**c**,**e**) Masking strategy S1 leads to maximal correct excitation but—due to occlusion and transparency—also to a small amount of incorrect excitation. (**d**,**f**) Strategies S2, S3 cause no incorrect excitations, but—due to occlusion—they reduce the level of correct excitations. The corresponding light-field masks are shown inset (approximated and optimized by solving Eq. 1). For illustration reasons, we applied 100 μm microspheres and a dry 20×/0.75 NA objective (see Table 1 for details) in the experiment for (**a–f**). See also Supplementary Video 2. (**g**–**i**) Optical axial-slice simulation of illumination strategies S1, S2 and S3 for eight excitation regions (*E*) and eight non-excitation regions (*N*). S1 (**g**) illuminates *E* (i–viii) while ignoring unwanted excitation of *N*, thus a high number of *N* (ix–xvi) is excited. S2 (**h**) avoids any unwanted excitation of *N* by sacrificing excitation light at *E* (e.g., regions ii and vi receive less light than in S1). S3 (**i**) avoids unwanted excitation of non-occluded non-excitation regions. Thus, *N* regions at the top of the volume (i.e., x, xiv, xv and xvi) are not excited while regions at the bottom of the volume (i.e., ix, xi, xii and xiii) receive light.

These three strategies are illustrated in Fig. 3 using the example of two occluding microspheres i and ii (Fig. 3a,b), and an optical axial-slice simulation of eight excitation regions and eight non-excitation regions (Fig. 3g–i). In Fig. 3c,d, we selected the foreground sphere i for excitation (i.e., to be part of *E*) and background sphere ii to remain unexcited (i.e., to be part of *N*), and vice versa in Fig. 3e,f. By choosing strategy S1, *E* receives a maximum of light while—due to partial occlusion and transparency of the microspheres/excitation regions—*N* is also fractionally illuminated (Fig. 3c,e,g). Strategy S2 avoids illumination of *N* entirely, but—depending on the degree of occlusion—it may result in lower excitation levels in *E* (Fig. 3d,f,h). Both strategies assume that the probes to be exited are not fully opaque but transmit a certain amount of excitation light. If probes can be considered to block light, then strategy S3 is appropriate (Fig. 3i). For our example in Fig. 3b, illuminating the nonoccluded microsphere i would result in the same outcome as shown in Fig. 3c (S1), while exciting the occluded microshpere ii results in Fig. 3f (S2). While computing an expensive optimization based on Eq. 1 (results are also shown in Fig. 3 for comparison) requires up to several hours (including generation of **B**), our raycasting approximation is achieved easily at interactive rates (see Method section).

## Discussion

According to Sparrow’s criterion, the smallest resolvable spot for wavelength *λ*, numerical aperture NA, and magnification *M* is 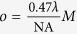. For a light-field microscope, the theoretical number of resolvable directions *D* equals the microlens pitch *m* divided by *o*. However, if the pixel pitch *p* of the SLM or camera used exceeds *o*, then *D* is limited to *m/p*. The lateral resolution in the field plane corresponds to the resolution of the MLA. The axial depth of field is *S* ≈ ((2 + *D*)*λn*)/(2NA^2^), which corresponds to the smallest focus shift within a focusable range of *F* ≈ ((2 + *D*^2^)*λn*)/(2NA^2^), where *n* is the refractive index of the imaging medium between the probe and the objective front lens (e.g., air *n* = 1, water *n* = 1.33, or oil *n* = 1.52)^5^. The numerical aperture of the microlenses should match the numerical aperture of the objective (F-number is *M*/(2NA)^5^).

**Figure 4:**
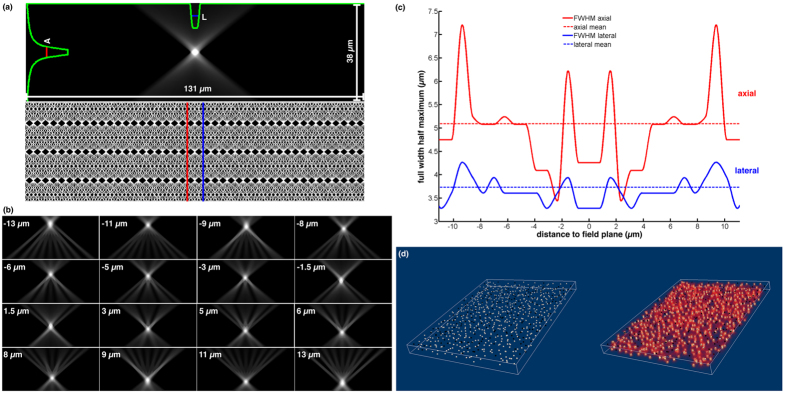
Optical simulation of light-field excitation sampling with a 40×/0.95 NA objective, a *m* = 125 μm microlens pitch, and a *λ* = 470 nm excitation wavelength. (**a**) PSF of light focused on the field plane and principal directions in light-field ray space. Note, that the volume is higher for illustration purposes and only covers half the FOV to avoid empty space. (**b**) PSF of light focused at varying distances to the field plane. (**c**) Lateral (blue) and axial (red) dimensions of focus points located at various distances along the red/blue lines in the ray space diagram shown in (**a**). (**d**) Volumetric light-field excitation example with strategy S2. White points are excitation regions (*E*). Black points are non-excitation regions (*N*). See Supplementary Video 3.

Figure 4 illustrates the sampling nature of our approach based on an optical simulation of an empty volume (i.e., without occlusions) within a lateral field of view of 263 μm and an axially focusable range *F* = 22.2 μm, achieved with a 40×/0.95 NA objective, a *m* = 125 μm microlens pitch, and a *λ* = 470 nm excitation wavelength. The point-spread function (PSF) of the illumination focused on the field plane is shown in Fig. 4a. The average full width at half maximum (FWHM) over all axial distances is approximately *L* = 3.75 μm (lateral) × *A* = 5.1 μm (axial), and corresponds to the smallest focusable point size (i.e., highest sampling resolution) in this example. The smallest axial focus shift is *S* = 2.9 μm. Since the light-field ray space (principal ray directions are illustrated in Fig. 4a) is discrete with a limited directional and spatial resolution, the ray-sampling density varies across the focusable range. The dimension of a focus point depends on the number of ray intersections at its position. Thus, the axial and lateral resolutions vary with the distance to the field plane, as shown in Fig. 4b,c. They range from 3.3 μm to 4.2 μm laterally and 3.5 μm to 7.2 μm axially for the example shown in Fig. 4. We are considering mean resolutions (dotted lines in Fig. 4c) in the remaining discussion.

While the lateral resolution of a light-field microscope can be increased mainly by downscaling the microlens pitch of the MLA, the axial resolution is enhanced by reducing the pixel pitch of the DMD (down to the diffraction limit set by *o*), increasing the NA of the objective, and reducing the excitation wavelength.

**Figure 5:**
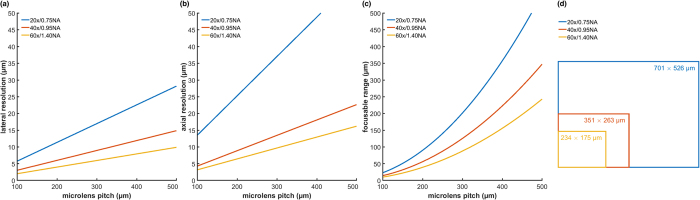
The impact of different microlens pitches *m* for dry objectives 20×/0.75 NA and 40×/0.95 NA, and for an oil immersion objective 60×/1.40 NA. (**a**) The mean lateral FWHM and (**b**) the mean axial FWHM of the PSFs determined by optical simulation indicate the lateral (*F*) and axial (*A*) resolutions. (**c**) The focusable range *F* and (**d**) the field of view for each objective. The simulated excitation wavelength was *λ* = 470 nm and the pixel pitch of the SLM *p* = 13.7 μm.

Figure 5 illustrates the impact of different microlens pitches (*m*) for three different objectives. While decreasing *m* increases the mean lateral and axial resolutions (*L, A*), the focusable range (*F*) is strongly reduced. The simulations in Fig. 4 uses a 40×/0.95 NA objective with a *m* = 125 μm microlens pitch. Thus, the following sampling is achieved: *o* = 9.3 μm, *p* = 13.7 μm, *D* = 9.12, *S* = 2.9 μm, *F* = 22.20 μm. The simulated PSF size for this configuration is lateral *L* = 3.75 μm and axial *A* = 5.1 μm, which results in a sampling density of 13.94 × 10^−3^ PSFs per μm^3^ within the excitable volume. The same 40×/0.95 NA objective with a *m* = 150 μm microlens pitch results in the following sampling parameters: *o* = 9.3 μm, *p* = 13.7 μm, *D* = 10.9, *S* = 3.37 μm, *F* = 31.74 μm. The PSF size for this configuration is lateral *L* = 4.5 μm (4.0–5.2 μm) and axial *A* = 7.5 μm (6.4–10.4 μm), which results in a sampling density of 6.63 × 10^−3^ μm^−3^.

**Figure 6:**
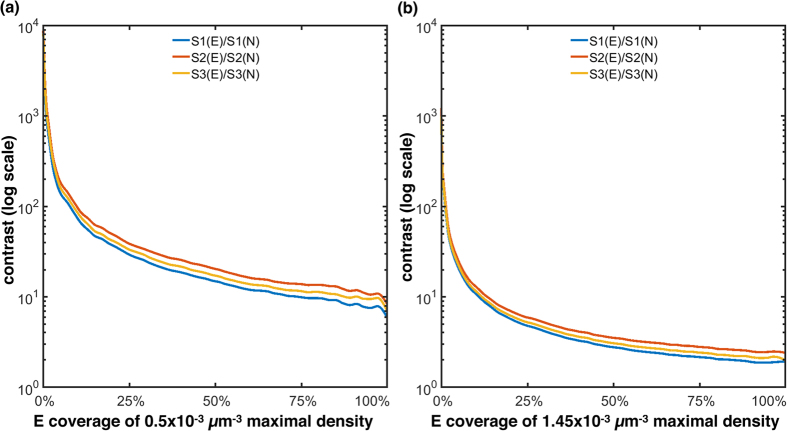
Simulated contrast levels for increasing numbers of points to be excited with the three masking strategies (S1, S2 and S3) and two settings. (**a**) 5.1 μm sized points, distributed in a volume of 263 × 351 × 22.2 μm^3^ at a maximal density of 0.5 × 10^−3^ μm^−3^, excited with a 40×/0.95 NA objective and *m* = 125 μm microlens pitch; (**b**) excitation with *m* = 150 μm microlens pitch (40×/0.95 NA objective) in a volume of 263 × 351 × 31.7 μm^3^, with 7.5 μm sized sample points at a density of 1.45 × 10^−3^ μm^−3^. Points are randomly distributed within the volumes and contrast is calculated by dividing the average illumination at *E* by the mean illumination at *N*. The volume is considered transparent and non-scattering. See Supplementary Video 4.

**Figure 7:**
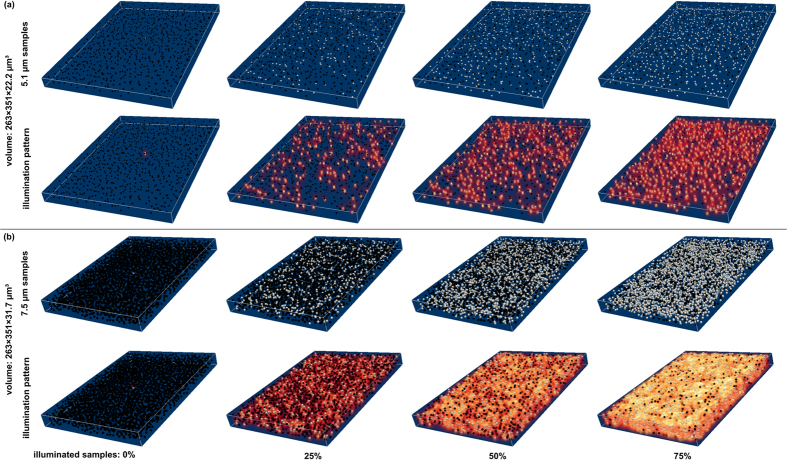
Simulated volumetric light-field excitation for an increasing numbers of sample points to be excited with masking strategy S2 and for the probe settings shown in Fig. 6. (**a**) Volume density of 0.5 × 10^−3^ μm^−3^, excited with a 40×/0.95 NA objective and *m* = 125 μm microlens pitch; (b) excitation with *m* = 150 μm microlens pitch (40×/0.95 NA objective) in a volume with density 1.45 × 10^−3^ μm^−3^. The top rows show the simulation volume with selected *E* (white) and *N* (black) sample points. The bottom rows visualize the sample points with resulting volumetric illumination pattern. Samples are randomly distributed within the volumes. The volume is considered transparent and non-scattering. See Supplementary Video 4.

Figure 6 illustrates the excitation contrast for the three masking strategies (S1, S2 and S3) and a varying number of excitation points (*E*) and non-excitation points (*N*). The points are randomly distributed based on a Poisson disk sampling^27^. Figure 6a shows an optical simulation with a density of 0.5 × 10^−3^ μm^−3^, while Fig. 6b simulates densities of 1.45 × 10^−3^ μm^−3^. For all three strategies the contrast decreases as the number of excitation positions *E* increases (consequently the number of *N* decreases). A high contrast indicates low light pollution at *N* and high excitation levels at *E*. Within transparent non-scattering volumes, strategy S2 generates the highest and S1 the lowest contrast. The simulation volumes and the resulting volumetric illumination patterns for strategy S2 are illustrated in Fig. 7a,b.

Our approach is currently limited to transparent and non-scattering probes, due to the usage of single-photon illumination. In the future we would like to investigate the combination of our method with two-photon excitation to support applications in dense scattering volumes. However, single shot two-photon light field acquisition is not possible. Therefore, alternative methods for focal stack recording—preliminary to volumetric two-photon light-field excitation—have to be employed. Furthermore, for two-photon excitation light efficient optics and high-power lasers are required.

Our experimental (Fig. 2) and simulated results (Figs 6 and 7) indicate that our technique might have potential applications in the field of optogenetics^28^,^29^. Typical neuron sizes and cell densities for model organisms—i.e., C. elegans and zebrafish larvae—are in the range of 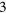
–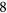 μm and 0.15–6.44 × 10^−3^ μm^−3^, respectively. However, currently we neglect some effects occurring in organic probes, such as scattering, natural fluctuations in cell size and density, and the neuron’s activation sensitivity. Future experiments on actual organic tissue will provide further insights.

## Method

Our proof-of concept prototype is a replica of the light-field microscope from Levoy *et al*.^22^ and is based on a Nikon Eclipse 80i fluorescence microscope. Our prototype equips a 20×/0.75 NA and a water-immersion 60×/1.2 NA objective (*n* = 1.33). For controlled and repeatable experiments, we applied commercially available fluorescence microspheres (100 μm and 10–20 μm) and bedded them in a silicone elastomer carrier. The application to other scales requires different MLAs or objectives and is discussed in the optical simulations above.

**Figure 8:**
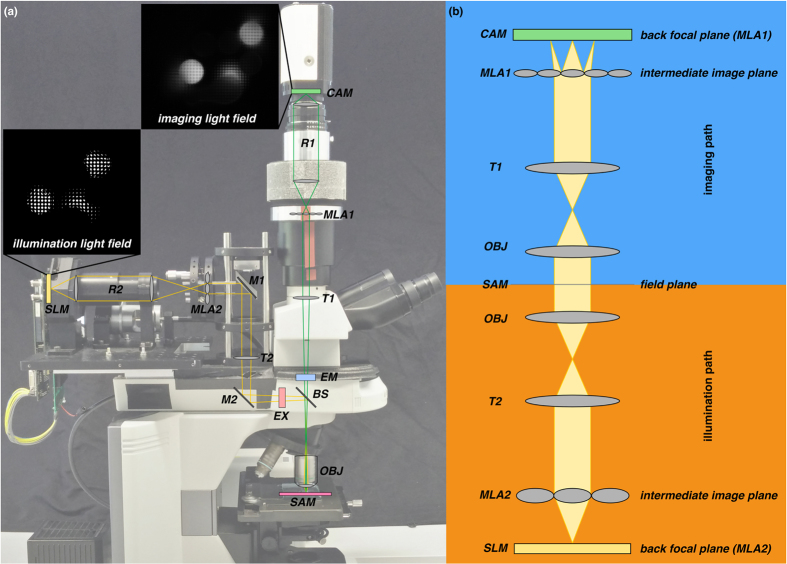
Light-field microscope prototype and optical trains used for imaging and illumination. (**a**) Light from the light source is conducted by a light guide and concentrated through an integrator rod and a TIR prism on the DMD-SLM (not shown in the figure, but explained by Levoy *et al*.^22^). The image of the DMD is focused on the back focal plane of the illumination microlens array MLA2 by a 1:1 telecentric relay lens R2 (Brilliant Technologies). Integration of light-field illumination into the standard microscope light port is achieved with the mirrors M1 and M2 and the tube lens T2 (focal length: 200 mm). For fluorescence applications, the excitation (EX) and the emission filters (EM) are placed in the standard Nikon microscopic filter cube, together with the beam splitter (BS). The illumination light field is focused by the objective (OBJ) on the sample (SAM). Light from the sample (SAM) passes through the objective (OBJ), the filter cube (BS and EM) and the tube lens (T1) (focal length: 200 mm) to the imaging microlens array (MLA1), which focuses it at the back focal plane of MLA1, where it is collected by the 1:1 relay lens system (R1, 2× Nikon AF Nikkor with nose-to-nose mounting, focal length: 50 mm) and imaged by the camera (CAM). The marginal rays for one imaging microlens (green) and one illumination microlens (yellow) are shown. (**b**) Simplified schematic of optics showing imaging and illumination rays formed by MLA1 and MLA2 for the case that the sample (SAM) is a surface mirror.

The prototype and the optical layout are shown in Fig. 8. The illumination system employs a square-sided, planoconvex microlens array (MLA2) at the intermediate image plane and a DMD-SLM (Texas Instruments Digital Light Processor driven by a Discovery 1100 Controller from Digital Light Innovations, pixel size *p*_*il*_ = 13.7 μm) with a resolution of 1024 × 768. As a light source we use a 120 W metal halide lamp (X-Cite 120, EXFO), with corresponding filters. For capturing light fields, the imaging system uses an MLA (MLA1) at the intermediate image plane. A monochrome CCD camera (Retiga 4000R, pixel size *p*_*il*_ = 7.4 μm) with 2048 × 2048 resolution is used for recording. The non-square illumination area, caused by the DMD, crops the usable field of view. For our experiments in Fig. 2 we used the 60 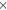 /1.2 NA objective and for the results in Fig. 3 we use the 20×/0.75 NA objective. The achieved sampling parameters with these configurations are shown in Table 1. The illumination loss of our prototype setup from light source to probe is approx 90%.

Eq. 1 assumes optical symmetry between the imaging and illumination light fields. In practice, however, slight misalignments in the optical paths and different microlenses cause misalignments and multi-mappings between illumination and imaging rays. To consider this, we calibrate the mapping between both by one-time measurement of a transport matrix **T** between the illumination light field **r**′ and the imaging light field **r** using a front surface mirror (with a 0.17 mm cover slip on top, placed perpendicular underneath the objective) using structured-light calibration^30^. The matrix **T** contains in each column the transport coefficients for one illumination ray **r**′_*s*,*t*,*u*,*v*_ to all imaging rays **r**_*s*,*t*,*u*,*v*_. Thus, the size of **T** equals the number of illumination rays (number of columns) times the number of imaging rays (number of rows). Note that, in order to compensate for the calibration with a front surface mirror, the angular coordinates of the illumination rays are inverted (i.e., **r**′_*s*,*t*,*u*,*v*_ becomes **r**′_*s*,*t*,−*u*,−*v*_) before their coefficients are stored in **T**. Eq. 1 then extends to **v** **=** **BTr**′, assuming that the z-stack from which **B** is determined is defined in the imaging ray space of **r**. The same applies to our approximated masks: Light fields in imaging ray space can be mapped to illumination ray space (or vice versa) by multiplying with **T** (or with **T^T^**). Note that **T** is constant for a constant optical configuration and is invariant to focus changes of the microscope. In the masking experiments with our 20× and 60× prototype **B** is large and contains 1.4 10^12^ and 4.31 10^11^ coefficients, respectively.

Our prototype implementation uses custom software^5^ to drive the microscope, Matlab for processing tasks (i.e., deconvolution, solving Eq. 1) and ImageJ for volume segmentation (i.e., selecting *E*). In Fig. 2 the z-stack size is 55 × 45 × 61 and sparse (i.e., a high number of volumetric points *i* is zero). For this setup our approximate solution of Eq. 1 was calculated in 7–10 seconds on an Intel Core i7 CPU 2.7 GHz and 24 GB of RAM. Computation times are independent of the number of non-zero volumetric points. In our approximation the illumination light field is constructed by raycasting its rays *j* in the volume (z-stack) and applying the rules of the masking strategies (S1, S2 and S3) as discussed above. Note, that the resulting light field is binary—0 for no light and 1 for light. We estimate that a GPU implementation of our approximation can lead to significant speed-up (at least ×10). When solving Eq. 1 with SART the number of non-zero volumetric points affects the performance. Runtimes ranged from 4 minutes for 0.16% non-zero points (*E* and *N*) up to 8 hours for 12.25% non-zero points. Note that most of the computation time is spend on construction of the transport matrix **B** (e.g., 8 hours for construction and 1 minute for solving with SART). The matrix **B** is constructed by raycasting each light-field ray *j* in the volume (z-stack) and setting the corresponding total transmission values *β*_*i*,*j*_ in the matrix. The computed illumination light field consists of continues values for 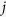. In our experiments we used 500 SART iterations and a single transmission value *α* for all non-empty volumetric points (*E* or *N*).

The samples in Fig. 2 were Fluorescent Green Polyethylene 10–20 μm microspheres (material density 0.99–1.01 g/cm^3^; peak excitation 470 nm; peak emission 505 nm; distributor cospheric) mixed with polydimethylsiloxane (Elastosil RT 604 from Wacker) of material density 0.97 g/cm^3^ (mixing ratio was 6.5% of spheres). In Fig. 3 we used Rhodamine B Polyethylene 100 μm microspheres (material density 0.98 g/cm^3^; peak excitation 540 nm; peak emission 630 nm; distributor cospheric) mixed with the same silicone elastomer (as above) at a mixing ratio of 5% spheres.

## Additional Information

**How to cite this article**: Schedl, D. C. and Bimber, O. Volumetric Light-Field Excitation. *Sci. Rep.* **6**, 29193; doi: 10.1038/srep29193 (2016).

## Supplementary Material

Supplementary Video Legends

Supplementary Video 1

Supplementary Video 2

Supplementary Video 3

Supplementary Video 4

## Figures and Tables

**Figure 1:**
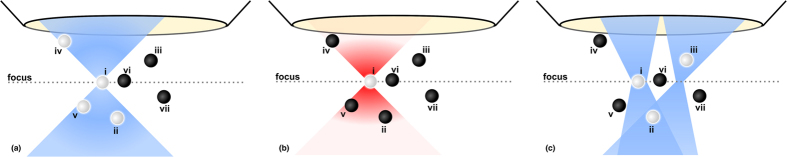
Excitation of microbeads i, ii and iii with different illumination techniques. (**a**) Spatial one-photon excitation of the in-focus microsphere i also excites spheres ii, iv, and v due to out-of-focus illumination. (**b**) With two-photon excitation only microshpere i at the focal plane emits light. (**c**) Spatial and angular beam control with volumetric light-field excitation irradiates only microspheres i, ii, and iii while avoiding illumination of the remaining probe.

**Table 1:** The sampling achieved with our prototypes equipped with 20×/0.75 NA and 60×/1.2 NA objectives and corresponding microlenses.

objective	imaging (MLA1)							illumination (MLA2)						
	*m*_im_	*D*_im_	*λ*_em_	*L*_*im*_	*A*_*im*_	*S*_im_	*F*_im_	*m*_il_	*D*_il_	*λ*_ex_	*L*_*il*_	*A*_*il*_	*S*_il_	*F*_il_
20×/0.75 NA	125	16 px	630 nm	7.8	17.7	10	141	300	22 px	540 nm	17.0	37.3	11.7	235
60×/1.2 NA	250	34 px	505 nm	5.3	9.4	5.4	112	300	22 px	470 nm	6.1	10.0	5.2	105

Units are in m if not stated otherwise.

## References

[b1] Bouchard M. B. . Swept confocally-aligned planar excitation (scape) microscopy for high-speed volumetric imaging of behaving organisms. Nat. Photonics 9, 113–119 (2015). 25663846 10.1038/nphoton.2014.323 PMC4317333

[b2] Quirin S., Peterka D. S. & Yuste R. Instantaneous three-dimensional sensing using spatial light modulator illumination with extended depth of field imaging. Opt. Express 21, 16007–16021 (2013). 23842387 10.1364/OE.21.016007 PMC3971059

[b3] Abrahamsson S. . Fast and sensitive multi-color 3d imaging using aberration-corrected multi-focus microscopy. Nat. Methods 10, 60–63 (2013). 23223154 10.1038/nmeth.2277 PMC4161287

[b4] Prevedel R. . Simultaneous whole-animal 3d imaging of neuronal activity using light-field microscopy. Nat. Methods 11, 727–730 (2014). 24836920 10.1038/nmeth.2964 PMC4100252

[b5] Levoy M., Ng R., Adams A., Footer M. & Horowitz M. Light field microscopy. ACM Trans. Graph. 25, 924–934 (2006).

[b6] Broxton M. . Wave optics theory and 3-d deconvolution for the light field microscope. Opt. Express 21, 25418–25439 (2013). 24150383 10.1364/OE.21.025418 PMC3867103

[b7] Cohen N. . Enhancing the performance of the light field microscope using wavefront coding. Opt. Express 22, 24817–24839 (2014). 25322056 10.1364/OE.22.024817 PMC4247191

[b8] Shepherd G. M., Pologruto T. A. & Svoboda K. Circuit analysis of experience-dependent plasticity in the developing rat barrel cortex. Neuron 38, 277–289 (2003). 12718861 10.1016/s0896-6273(03)00152-1

[b9] Guo Z. V., Hart A. C. & Ramanathan S. Optical interrogation of neural circuits in caenorhabditis elegans. Nat. Methods 6, 891–896 (2009). 19898486 10.1038/nmeth.1397 PMC3108858

[b10] Lutz C. . Holographic photolysis of caged neurotransmitters. Nat. Methods 5, 821–827 (2008). 19160517 10.1038/nmeth.1241 PMC2711023

[b11] Nikolenko V. . SLM microscopy: scanless two-photon imaging and photostimulation with spatial light modulators. Front. Neural Circuits 2, doi: 10.3389/neuro.04.005.2008 (2008). PMC2614319 19129923

[b12] Papagiakoumou E. . Scanless two-photon excitation of channelrhodopsin-2. Nat. Methods 7, 848–854 (2010). 20852649 10.1038/nmeth.1505 PMC7645960

[b13] Reutsky-Gefen I. . Holographic optogenetic stimulation of patterned neuronal activity for vision restoration. Nat. Commun. 4, 1509 (2013). 23443537 10.1038/ncomms2500

[b14] Packer A. M., Russell L. E., Dalgleish H. W. & Häusser M. Simultaneous all-optical manipulation and recording of neural circuit activity with cellular resolution *in vivo*. Nat. Methods 12, 140–146 (2015). 25532138 10.1038/nmeth.3217 PMC4933203

[b15] Rückerl F. . Micro mirror arrays as high-resolution spatial light modulators for photoactivation and optogenetics. In SPIE BiOS 85860U–85860U (International Society for Optics and Photonics, 2013).

[b16] Rückerl F., Berndt D., Heber J. & Shorte S. Photoactivation and optogenetics with micro mirror enhanced illumination. In SPIE Photonics Europe 913017–913017 (International Society for Optics and Photonics, 2014).

[b17] Packer A. M. . Two-photon optogenetics of dendritic spines and neural circuits. Nat. Methods 9, 1202–1205 (2012). 23142873 10.1038/nmeth.2249 PMC3518588

[b18] Paluch-Siegler S. . All-optical bidirectional neural interfacing using hybrid multiphoton holographic optogenetic stimulation. Neurophotonics 2, 031208–031208 (2015). 26217673 10.1117/1.NPh.2.3.031208 PMC4512959

[b19] Golan L., Reutsky I., Farah N. & Shoham S. Design and characteristics of holographic neural photo-stimulation systems. J. Neural Eng. 6, 066004 (2009). 19837999 10.1088/1741-2560/6/6/066004

[b20] Vaziri A. & Emiliani V. Reshaping the optical dimension in optogenetics. Curr. Opin. Neurobiol. 22, 128–137 (2012). 22209216 10.1016/j.conb.2011.11.011

[b21] Zhang Z., Barbastathis G. & Levoy M. Limitations of coherent computer generated holograms. In Digital Holography and Three-Dimensional Imaging DTuB5 (Optical Society of America, 2011).

[b22] Levoy M., Zhang Z. & Mcdowall I. Recording and controlling the 4d light field in a microscope using microlens arrays. J. Microsc. 235, 144–162 (2009). 19659909 10.1111/j.1365-2818.2009.03195.x

[b23] Isaksen A., McMillan L. & Gortler S. J. Dynamically reparameterized light fields. In SIGGRAPH 297–306 (ACM, 2000).

[b24] Levoy M. . Synthetic aperture confocal imaging. ACM Trans. Graph. 23, 825–834 (2004).

[b25] Holmes T. J. . Light microscopic images reconstructed by maximum likelihood deconvolution. In Pawley J. B. (ed.) Handbook of biological confocal microscopy 389–402 (Springer, 1995).

[b26] Andersen A. & Kak A. C. Simultaneous algebraic reconstruction technique (sart): a superior implementation of the art algorithm. Ultrason. Imaging 6, 81–94 (1984). 6548059 10.1177/016173468400600107

[b27] Cook R. L. Stochastic sampling in computer graphics. ACM Trans. Graph. 5, 51–72 (1986).

[b28] Lima S. Q. & Miesenböck G. Remote control of behavior through genetically targeted photostimulation of neurons. Cell 121, 141–152 (2005). 15820685 10.1016/j.cell.2005.02.004

[b29] Boyden E. S., Zhang F., Bamberg E., Nagel G. & Deisseroth K. Millisecond-timescale, genetically targeted optical control of neural activity. Nat. Neurosci. 8, 1263–1268 (2005). 16116447 10.1038/nn1525

[b30] Sen P. . Dual photography. ACM Trans. Graph. 24, 745–755 (2005).

