# Supplementary material for: Volumetric Light-Field Excitation
Source: Sci Rep. 2016 Jul 1;6:29193. doi: 10.1038/srep29193 (PMC4929678; doi:10.1038/srep29193)
Supplement: Supplementary Video Legends [file srep29193-s1.pdf]

Supplementary Information: Volumetric Light-Field Excitation

David C. Schedl[1] and Oliver Bimber[1,*]

[1]Institute of Computer Graphics, Johannes Kepler University, Linz, 4020, Austria
*oliver.bimber@jku.at

Supplementary Video Legends

Supplementary Video 1: Volumetric excitation principle with 10–20 μm microspheres and 60×/1.2NA objective: light-field imaging of specimen under full illumination, synthetic aperture rendering to obtain 3D focal stack, 3D deconvolution to obtain 3D z-stack, segmentation of selected regions, projection of masked light-field illumination into the specimen. Comparison: full and masked illumination.

Supplementary Video 2: Example of illumination with computed light-field masks: Two occluding microspheres (front and back), where one is to be excited, while the remaining scene is to remain unexcited. Masking strategy S1 leads to maximal correct excitation but—due to occlusion and transparency—also to a small amount of incorrect excitation. Strategies S2, S3 cause no incorrect excitations, but—due to occlusion—they reduce the level of correct excitations. For illustration reasons, we applied 100 μm microspheres and a dry 20×/0.75NA objective.

Supplementary Video 3: Optical simulation of light-field excitation sampling with a 40×/0.95NA objective, a $m = 125$ μm microlens pitch, and a $\lambda = 470$ nm excitation wavelength: PSF of light focused at varying distances to the field plane. Volume excitation example with strategy S2. White points are excitation regions (E). Black points are non-excitation regions (N).

Supplementary Video 4: Optical simulation of volumetric excitation for increasing numbers of points to be excited with the masking strategies S2 for 5.1 μm sized points (distributed in a volume of $263 \times 351 \times 22.2$ μm^3 at a maximal density of 0.5×10^{-3} μm^{-3}, excited with a 40×/0.95NA objective and $m = 125$ μm microlens pitch), and for 7.5 μm sized points (excitation with $m = 150$ μm microlens pitch in a volume of $263 \times 351 \times 31.7$ μm^3, at density of 1.45×10^{-3} μm^{-3}). Points are randomly distributed within the volumes and contrast is calculated by dividing the average illumination at E by the mean illumination at N. The volume is considered transparent and non-scattering.
